# The Effects of Onychectomy (Declawing) on Antebrachial Myology across the Full Body Size Range of Exotic Species of Felidae

**DOI:** 10.3390/ani13152462

**Published:** 2023-07-30

**Authors:** Lara L. Martens, Sarah Jessica Piersanti, Arin Berger, Nicole A. Kida, Ashley R. Deutsch, Kathryn Bertok, Lauren Humphries, Angela Lassiter, Adam Hartstone-Rose

**Affiliations:** 1 Department of Biological Sciences, North Carolina State University, Raleigh, NC 27695, USA; llmarten@ncsu.edu (L.L.M.); spiersa@ncsu.edu (S.J.P.); snberger@ncsu.edu (A.B.); nakida@ncsu.edu (N.A.K.); adeutsc@ncsu.edu (A.R.D.); 2 Department of Biological Sciences, Arizona State University, Tempe, AZ 85281, USA; 3 Carolina Tiger Rescue, Pittsboro, NC 27312, USA; kathrynbertok@carolinatigerrescue.org (K.B.); laurenhumphries@carolinatigerrescue.org (L.H.); angelalassiter@carolinatigerrescue.org (A.L.)

**Keywords:** *Panthera*, tigers, exotic cats, wild cats, captivity

## Abstract

**Simple Summary:**

While people are familiar with the practice of declawing domestic cats, it is also performed on non-domesticated species, such as lions and tigers, to extend the period of safe human interaction for entertainment purposes. Although declawing (removing not only the claw itself, but also the bone from which it grows, whether in part or in whole) clearly affects the skeletal system, the impact on the musculature has not been studied. As the mass of an animal increases cubically as a product of its volume, while the areas of its paws only increase as a square, larger cats have higher foot pressures and, therefore, the surgery may have a greater effect on larger cats. In this study, we evaluate the forearms of clawed and declawed cats to investigate the effects of declawing on muscle architecture. We found that the deep muscles that flex the digits, which are the muscles most directly affected by declawing, are significantly lighter (~73%) and less powerful (46–66%) in animals that have been declawed, while other muscles do not make up for these reductions. Thus, declawing has a substantial effect on the muscular capabilities of cats, and because these deficiencies are not compensated for in larger cats, it probably has even more functionally devastating consequences for these species.

**Abstract:**

While people are familiar with the practice of declawing domestic cats, “onychectomy”, as it is also known, is also performed on non-domesticated species, including pantherines, to prolong their use for entertainment purposes. Although the surgery (the partial or complete removal of the distal phalanx) has clear osteological implications, its myological effects have never been studied. As the mass of an animal increases cubically as a product of its volume, while the areas of its paws only increase as a square, larger felids have higher foot pressures and, therefore, the surgery may have particularly substantial functional effects on larger cats. In this study, we evaluate the forearms of clawed and declawed non-domestic felid specimens that spanned the body size range of the whole family to evaluate the effects of onychectomy on muscle fiber architecture. We found that the deep digital flexors (the muscles most directly affected by onychectomy) of declawed felids are significantly lighter (~73%) and less powerful (46–66%) than those of non-declawed felids, while other muscles do not make up for these reductions. Thus, onychectomy has a substantial effect on the myological capabilities of cats, and because these deficiencies are not compensated for in biomechanically disadvantaged larger felids, it probably has even more functionally devastating consequences for these species.

## 1. Introduction

Onychectomy, i.e., the surgery in which the claw and nailbed or whole distal phalanx are removed to “declaw” carnivores [1,2,3], is performed on domestic cats throughout the United States [4,5,6], generally occurring at the request of cat owners who wish to minimize scratching and furniture damage caused by their pets [7]. These surgeries are also performed on exotic animals. In smaller non-domesticated animals (e.g., kinkajous and servals, i.e., *Potos flavus* and *Leptailurus serval*, respectively), the motivation for onychectomy is mostly the same as it is in domesticated cats—to reduce furniture damage or scratching during handling [3,8]. However, they are also performed on large cats (e.g., cougars and tigers, i.e., *Puma concolor* and *Panthera tigris*, respectively), predominantly as part of the cub-petting industry, in which clients pay to play and take photographs with baby animals before they grow too large for humans to safely interact with them [8,9]. While onychectomy has been banned in many countries for domestic cats [4,5], the procedure likely has much more damaging effects on larger animals. This belief is likely true because of the differential scaling of paw size and body mass. Indeed, across the size range of felids, paw area—a square variable—increases at a slower rate than body mass, which is proportional to volume—a cubic variable. Thus, larger cats have smaller feet relative to their body size, and their paws must withstand significantly greater amounts of pressure, leading to the biomechanical likelihood that declawing has a more anatomically devastating effect on larger species. In the current study of clawed and declawed cat specimens, which spans the whole-body size range of the Felidae family, we aim to document the impact that this surgery has on the muscles that both attach to the phalangeal elements modified during this surgery and may compensate for locomotor functions damaged during this surgery. Regardless of whether the surgery is performed solely on forelimbs or both forelimbs and hindlimbs, the anatomical effect is likely similar or more drastic in the forelimbs, as a greater portion of the body mass is supported by these limbs in felids [10,11,12]; thus, our study focuses exclusively on the myological effects of onychectomy on the forelimb.

### 1.1. The Motivation behind Onychectomies

Onychectomies are performed for a variety of reasons. In domestic cats, the majority of owners request that this procedure is performed on their cats to prevent scratching [7]. Destructive household scratching is the most common complaint that pet owners have about their pet cats [7,13]. Not only is scratching seen as a problematic behavior because it destroys property, it can also present a health risk to those in contact with the cat. For example, senior citizens and those who are immunocompromised or otherwise at a higher risk of disease or bodily harm related to cat scratches may elect to have their cats declawed in order to physically protect themselves [14]. Owners may feel that declawing to curb scratching is the only alternative they have to relinquishing or euthanizing their cat [7,15,16]. This attitude may not be unrealistic, as behavioral problems are the most common reason for the relinquishment or euthanasia of cats [7]. As owners who choose to have their cats declawed generally reported that the procedure met or exceeded their expectations [2,17], as well as reporting an improvement in the owner–cat relationship as a direct result of the declawing [2,14], owners may feel that this is an optimal treatment for problematic behaviors. Additionally, some states allow landlords to require that any cat on the property be declawed [5]. Therefore, declawing their cat may be the only way that owners can retain their pets. There are also some medical reasons for declawing cats, as declawing is the optimal treatment for certain diseases, such as paronychia and neoplasia, though these causes represent a minority of cases [18].

While domestic cats are most often declawed to prevent scratching and other problematic behaviors, non-domestic cats are most often declawed to make these dangerous animals easier to handle as pets, during public interactions, or while performing for public entertainment [3]. They are declawed—and also often surgically modified in other ways, such as via canine tooth extraction or, more commonly, filing—to make it more convenient to own these increasingly popular cats [8]. Certain facilities have also been known to declaw all of their big cat cubs prior to selling them [9]. These surgical modifications, in addition to drugging large cats, are predominantly used in industries where the public has contact with cubs of large cat species; this industries are often called “pay-to-play” and “pay-to-pet” [19], in which cubs, often lions and tigers (*Panthera leo* and *P. tigris* respectively), are removed from their mothers soon after birth and handled by members of the public from the ages of 8–12 weeks. Tiger cubs are especially vulnerable to this kind of activity, as the USDA has a special exemption that allows people to have direct contact with tiger cubs [9].

### 1.2. Rates and Legality of Onychectomies

Declawed domestic cats, while representing a minority of domestic cats, are not uncommon. Studies have shown onychectomy rates of 20 to 45% in the USA [20,21,22,23]. The specific prevalence of declawed non-domestic cats is not available; however, as many non-domestic cats are kept in unregulated zoos and private homes [24], there are many non-domestic cats at risk of being declawed.

Due to concerns about the ethicality of declawing and its prevalence, there have been movements to outlaw the surgery. Onychectomies have never been particularly popular outside of the United States, as much of the world considers them to be cruel. In Australia, onychectomies are regulated at the state level, though the practice is banned in nearly every state. The Canadian Veterinary Medical Association condemns the practice [5], and it is outlawed in some areas of the country [4]. In Europe, the Protection of Pet Animals treaty, which was signed in 1987, banned onychectomies in all signatory nations, including Austria, Belgium, Bosnia, Denmark, Finland, France, Germany, Ireland, Italy, Malta, Netherlands, New Zealand, Northern Ireland, Norway, Portugal, Scotland, Serbia, Slovenia, Sweden, Switzerland, and Wales; this surgery is also outlawed in Brazil and Israel [5].

Although there have been a variety of movements that proposed outlawing onychectomies in the US [6], the procedure remains legal in most states, being illegal only in Maryland and New York [4,5]. Some US cities have also outlawed onychectomies, including six cities in California [4,5]. Additionally, many individual clinics and veterinarians refuse to perform the procedure, and animal welfare groups continue to advocate for the banning of onychectomies for domestic cats in general [5].

Onychectomies are more widely banned in non-domesticated cats due to the extra protection afforded to these groups. Federally, non-domesticated cats are protected against declawing by the US Department of Agriculture (they are covered as “captive wild or exotic carnivores”) under the Animal Welfare Act, though this protection is not complete and still permits declawing if recommended by a veterinarian. Additionally, California, Indiana, and Michigan have banned declawing non-domestic cats unless required to treat a legitimate medical concern. Under the Endangered Species Act, certain non-domestic cat species (those that are considered in danger of extinction) are protected from harassment and harm. This regulation covers declawing, as decided by the court in PETA v. Wildlife in Need and Wildlife in Deed, Inc. However, the practice is still widespread, and it has anatomical implications beyond simply the removal of the claws [5].

### 1.3. Antebrachial Anatomy

**Figure 1:**
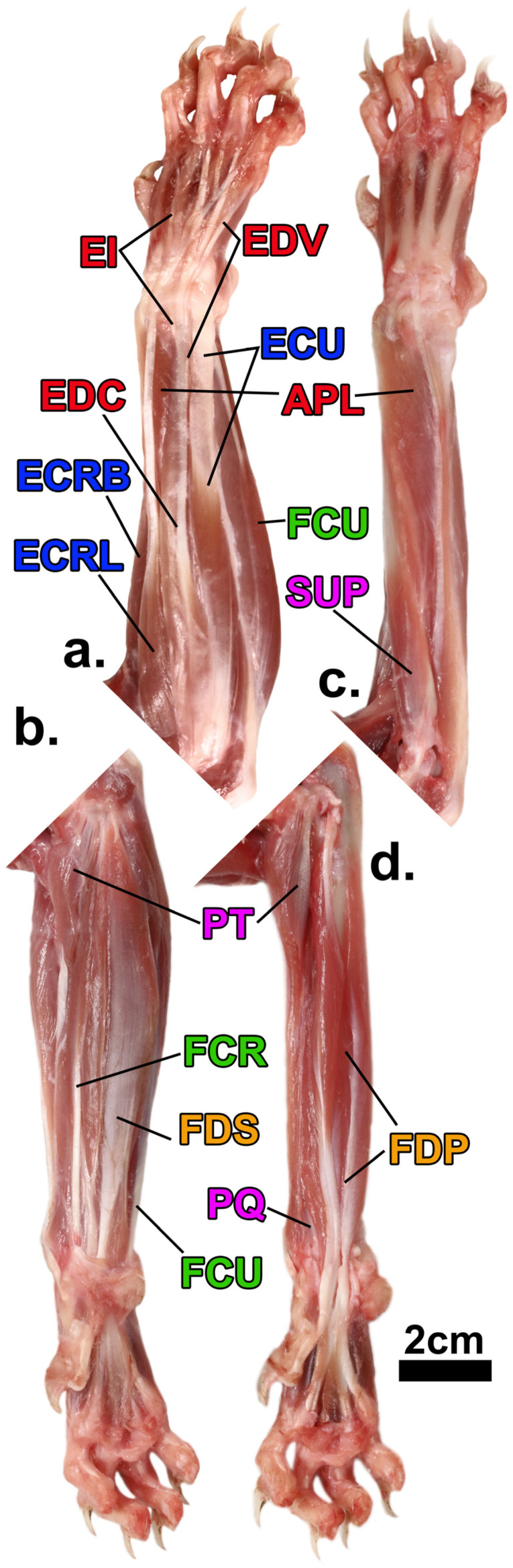
Forearm muscles of *Felis catus*. (**a**,**b**) show superficial images of the anterior (flexor) and posterior (extensor) compartments, respectively; (**c**,**d**) show superficial images of deeper muscles of the same compartments of the forearm. Abbreviations: EI, m. extensor indicis (attaching to ray II only in this specimen); EDV, m. extensor ray V; ECU, m. extensor carpi ulnaris; APL; m. abductor pollicis longus; EDC, m. extensor digitorum communis; FCU, m. flexor carpi ulnaris; ECRB, m. extensor carpi radialis brevis; ECRL; m. extensor carpi radialis longus (ECRB and ECRL are differentiated based on insertion and length) [25,31]; SUP, m. supinator; PT, m. pronator teres; FCR, m. flexor carpi radialis; FDS, m. flexor digitorum superficialis; FDP, m. flexor digitorum profundus–humeral head; PQ, m. pronator quadratus; FCU, m. flexor carpi ulnaris. Colors of the functional groups are designated as follows: Yellow = digital flexors, green = wrist flexors, red = digital extensors, blue = wrist extensors, and pink = pronators/supinators. Although this specimen was dissected for illustrative purposes, only non-domesticated species were included in our analyses.

**Figure 2:**
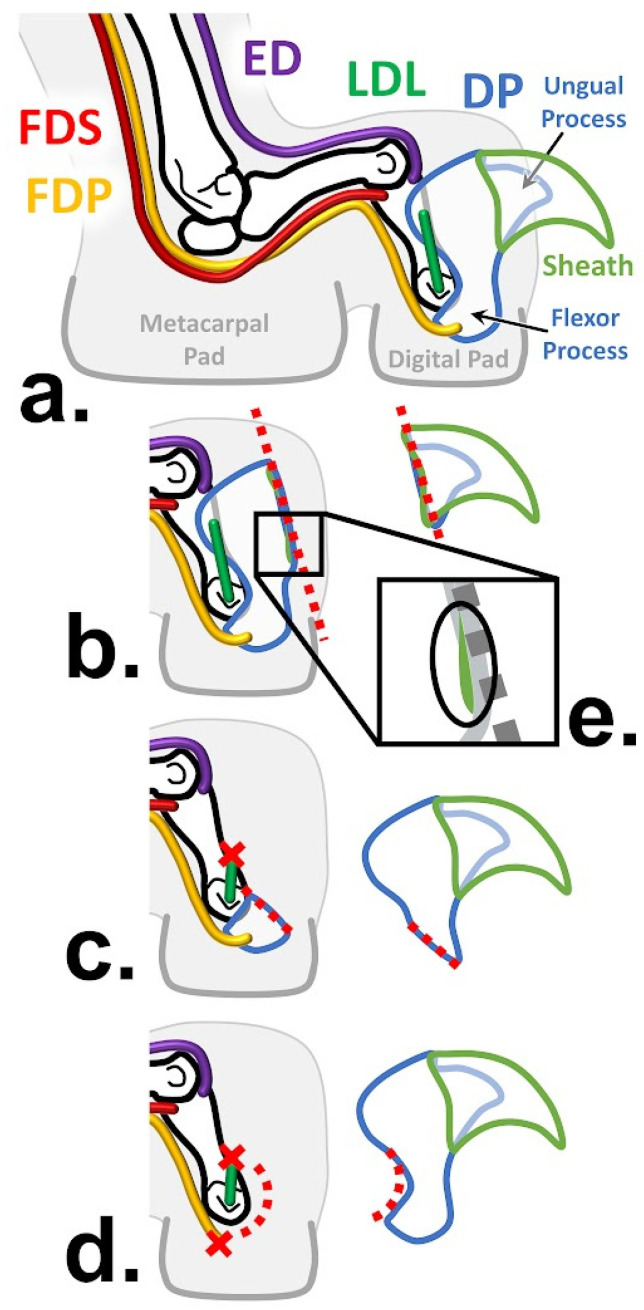
Schematic illustration of felid retractile claw anatomy and variations in onychectomy. (**a**) Unaltered anatomy adapted from the study of Bryant et al. (1996) [29]. Onychectomy via (**b**) removal of the ungual process and claw sheath, (**c**) removal of the distal phalanx (DP) through severance of the flexor process and lateral dorsal elastic ligament (LDL), or (**d**) removal of the whole DP through accompanying severance of the LDL and flexor digitorum profundus (FDP) tendon; these methods were adapted from Clark et al.’s schematic drawing (2014) [1]. Notably, in the ungual process and claw sheath removal approach (**b**), claw propagating tissue may be left behind (**e**), leading to claw regrowth. Red dotted lines and X’s indicate the approximate surgical plane and structures to be surgically severed, respectively. Abbreviations: FDS, m. flexor digitorum superficialis; FDP, m. flexor digitorum profundus; ED, extensor digitorum communis.

While onychectomy may affect the anatomy of the entire limb, the distal elements—the wrist and ankle bones, the digital rays, and the muscles that fire them—most of which are found in the forearm and leg, respectively, are likely to be most affected by the surgery. The forearm muscles, which are responsible for movement of the wrist and digits, can be categorized into six functional groups (Figure 1): forearm pronation, forearm supination, wrist extension, wrist flexion, digital extension, and digital flexion [25,26]. Wrist and digital flexors are of particular interest when examining the potential impacts of onychectomy as, when combined with ligament and tendon support, flexors of the wrist and digits yield the necessary rigidity required to maintain digitigrade posture (the stance via which body weight is transmitted to the ground entirely through the toes) and locomotion under a full spectrum of demands, including high-impact loads from landing large jumps, without the risk of posture collapsing to the palmar surface [11,27,28]. Among these flexors, flexor digitorum profundus (FDP) is perhaps the most pertinent, as it inserts into the distal phalanx, which is the target of declawing surgery. As in most mammals, the felid digital rays II-V are comprised of proximal, intermediate, and distal phalanges, while ray I lacks an intermediate phalanx [29]. Affixed to the end of each distal phalanx is an ungual process that acts as an internal support for the sharp and curved keratin claws Figure 2a [1,29,30].

At rest, the clawed distal phalanges of rays II-V are held in a passively retracted position on the lateral side of the intermediate phalanx by the dorsal elastic ligaments [29,30]. While independent contraction of FDP produces digital flexion at the distal interphalangeal joint (DIP), thus curling the phalangeal elements toward the palmar surface [25], it also works in concert with flexor digitorum superficialis (FDS) and digital extensors to yield claw protraction [30]. When the digital extensors co-activate with FDP, they act as a stopper to prevent flexion of the wrist and hand, instead allowing the force to aid FDP in overcoming the retractive resistance of the dorsal elastic ligaments to protract the claws [30]. As four functional movements occur at the DIP joint (claw protraction, claw retraction, digital extension, and digital flexion), the collateral ligaments are needed to provide stability by preventing lateral movement during these extension- and flexion-based movements [2,32].

### 1.4. Forelimb Function in Felids

In felids, the claws and distal phalanges not only allow grasping during hunting and feeding, but also the maintenance of static posture and movement. Cats condition their claws through scratching behaviors to maintain length and sharpness [33], as claws are essential for defense, climbing, communicative marking, and prey acquisition. Felids use their sharp and curved claws to penetrate the surface of trees and other vertical substrates while climbing, especially those species, such as the clouded leopard, ocelot, and margay (*Neofelis nebulosa, Leopardus pardalis*, and *L. wiedii,* respectively), that are relatively arboreal among felids [34,35,36,37]. Trees and other surfaces are also used for scratching as a means of depositing chemicals excreted by interdigital glands for territorial marking and other intraspecies communication [33,38]. When hunting, cats protract their claws to pounce, grasp, hold, and grapple prey into submission before administering lethal action with their jaws [12,30]. Cats that hunt relatively large prey are particularly reliant on robust and powerful forelimbs and claws to contend with struggling prey, while small prey specialists require longer forelimbs that possess flexibility that allows sufficient supination and digital flexion to subdue quick-moving prey [12,39]. Distal phalanges are also an integral component of digitigrade posture and locomotion.

### 1.5. Onychectomies as a Surgery

As the claws and distal phalanges facilitate a wide array of behaviors, their amputation modifies the structure and function of the forelimb. Onychectomy, which is colloquially referred to as “declawing”, is an elective surgical procedure commonly performed on felids, during which the claws are removed, and it oftentimes involves the partial or complete amputation of the distal phalanx (Figure 2) from which the claws originate [1,2,3]. Declawing is generally accomplished via either a “disarticulation method”, i.e., using a laser or scalpel, or a “guillotine method”, i.e., using a nail trimmer or wire saw for small or large cats, respectively [1,3,40]. The surgical line is determined based on the desired level of amputation: isolated removal of the claws at the ungual process (Figure 2b), partial amputation of distal phalanx leaving the flexor process in situ (Figure 2c)*,* or complete amputation of the entire distal phalanx Figure 2d [1,3]. During complete amputation, the deep digital flexor tendons, dorsal elastic ligaments, and collateral ligaments are all severed to disarticulate the DIP joint [2]. In partial amputation, in which the flexor process is left in situ (Figure 2c), the risk of post-surgical claw regrowth and irritation from sharp bony remnants increases; however, FDP’s tendon and function are preserved [1,3,40]. Although independent claw removal at the ungual process (Figure 2b) preserves all DIP joint structures and functions, it is the least commonly performed onychectomy method and carries the highest risk of claw regrowth, as it is difficult to ensure that all claw-propagating tissue has been removed (Figure 2e) [3].

There are a variety of complications associated with onychectomies, which can have either a long- or short-term nature, for species of all sizes. Onychectomies interfere with the natural anatomy and behavior of animals [8], though these complications may be difficult to observe due to the stoic nature of cats [5]. The prevalence of reported complications ranges from 3–50% [41], and, in both domestic and non-domestic felid species, complications include hemorrhaging, infection, neurapraxia, loss of the digital pad, incomplete healing, exposure of the second phalanx, claw regrowth, and tissue necrosis [42]. Wound reopening [14], distal limb ischemia [14], laceration of the digital pad [3], and paralysis of the limb [3] are also possible. Domestic cats have also been reported to demonstrate additional short-term complications, such as defecating and urinating on the floor—behaviors regarded as potential responses to pain [5]. This issue can also worsen chronic conditions seen in house cats, such as skin disorders, asthma, and cystitis [14]. While these examples represent short-term complications, there are also many long-term complications [43]. These complications include long-term paralysis, bilateral tendon flexure, fibrosis, and adhesions in the tendons and soft tissue that surround the intermediate phalanx [14]. In non-domestic cats, the lack of appropriate or sufficiently large surgical tools during onychectomies [44] may lead to additional or worse complications than those seen in house cats. Excessive post-surgical licking has also been observed in non-domestic cats; while some paw licking is considered a normal response to inflammation after surgery, persistent paw self-mutilation (licking until wounded and chewing on paw) has been observed and indicates more than typical post-surgical inflammation [3].

The change in the anatomy of declawed cats has additional ambulatory implications, such as arthritis, paw pad disruption, and abnormal stance and gait [45]. If the onychectomy is improperly performed or the distal phalanx is not completely removed, there is a possibility of claw regrowth and scur formation [3]. This issue can further limit the declawed cat’s movement as the animal is forced to either walk on the painful bone fragment, causing “pebble-in-the-shoe”-like pain and discomfort, or avoid walking on its digits to prevent this pain, instead walking on the carpus and tarsus [45], essentially changing from a typical felid digitigrade stance to a plantigrade stance found in, for instance, primates and bears, but never non-pathological felids. This issue has regularly been observed in non-domestic felids as they reach weights of over 200 kg, worsening their gait over time and leading to back problems, along with arthritis and ulcers [8]. Also common in declawed non-domestic cats is a syndrome known as “floppy-foot”, which causes cats to struggle to properly flex and extend their paws, forcing them to walk in a flat-footed manner [3,45].

### 1.6. The Allometric Problem

Within the Felidae family, there is a great diversity of body sizes that spans more than two orders of magnitude. The smallest of cats, *Prionailurus rubiginosus*, weighs as little as 1.1 kg, while the largest of cats, *Panthera tigris*, can weigh over 300 kg [46]. However, the scaling differences between volume and surface areas cause an allometric problem: as mass is a cubic variable (it is directly proportional to volume) and surface area is a square variable, animal body mass increases more substantially in larger animals than paw surface area. This observation means that larger felids have smaller paws relative to their body size, presenting a substantial pressure difference.

It is, however, clear that large felids are adapted to this increase in pressure. Despite the theoretical strain on the soft and hard tissues, they are capable of normal motion and gait [10,47,48]. To accommodate the need for greater propulsive force, the physiological cross-sectional area (the myological proxy for force production) of forelimb muscles may scale positively with increasing body mass, while the other limb muscles scale with negative allometry (i.e., large cats have relatively weaker shoulders [10]). However, the supporting muscles of the shoulder, such as serratus ventralis cervicis and trapezius thoracis, contract slower and more forcefully to improve support and compensate for the relatively smaller muscles in this area of larger felids [10]. To support the differences between muscles and the increased strain, felids have adapted skeletal differences with increasing size: as they increase in size, their skeletons increase in robustness to sustain these loads. Variable scaling of bone size and thickness within forearm bones allows larger forearm muscle attachments as felids increase in size, as well as negating the impact of the increased strain [49]. The muscles of larger felids also have more extensive mechanical advantages due to smaller angles and energy storage in elastic tendons, which cause more strain on the tendons while enabling more efficient running and movement [50]. These adaptations also explain how felids can maintain a similar posture across sizes—unlike the typically more erect posture and bone arrangement seen in larger mammals in general—while maintaining a high mechanical advantage [10,48]. Further evidence that felids have evolved to accommodate this scaling problem is the fact that larger felid paws have mechanically adapted to handle the differences in strain. Indeed, their paw pads are stiffer and store additional elastic energy in their tendons [47].

However, when the forearm muscle tendons that provide additional elastic energy are severed during onychectomies, both the active muscle and passive tendon compensatory abilities are potentially diminished or completely destroyed. Thus, although in their clawed and unaltered state, larger felids are clearly adapted to handle this allometric problem of higher body weight relative to paw surface area, declawing may have a more detrimental effect on the limb musculature in larger felids. Additionally, these impacts may be most significant in the forelimbs and paws, as they support up to 60% of body weight [10]. Combined with other factors, such as the extreme increase in growth and body mass of larger felids from their juvenile to adult size and the fact that onychectomies are usually performed on felids when they are very early in their post-natal development [9,19], increasing potential ontogenetic and functional effects of this modification may mean that non-domestic cats myologically suffer more greatly as a result of onychectomies.

## 2. Hypotheses

To this end, we propose to test the following hypotheses:

**H1.**  *Across the sample, felid forearm muscle mass (MM), physiological cross-sectional area (PCSA), and fascicle length (FL) will scale with isometry or positive allometry, as has been typically found in other myological systems and the forearms of other lineages [51,52,53]. A finding of positive allometry of MM and/or PCSA may be expected because of the allometric problem (the different scaling of paw area and body mass necessitates greater myological abilities in larger animals), while the fact that large cats hunt prey of a relatively large size compared to their body mass also potentially necessitates larger force-producing abilities [10,12].*

**H2.**  *Due to the allometric problem, the differences between clawed and declawed felids in MM, PCSA, and FL will be more extreme in relatively larger felids.*

**H3.**  *As tendons for the digital flexors are severed during a partial or whole amputative onychectomy [7,14] (Figure 2), the muscles most associated with onychectomy may be atrophied and, therefore, smaller and less powerful. Previous research in rats has shown muscle atrophy due to tendon tears [54]. Digital flexor forearm muscles of declawed felids will have relatively lower MM and PCSA.*

**H4.**  *If onychectomy reduces the mass and/or PCSA of the digital flexors, the associated reduction in the function of the phalanges may impact the function of the forearm as a whole, reducing the capacity of the other forearm muscles, leading to relatively small wrist extensor and flexor MM and PCSA in declawed specimens (H4a). Alternatively, the wrist extensor and flexor muscles may compensate for the reduced digital muscle capacity, leading to relatively large wrist extensor and flexor MM and PCSA in declawed specimens (H4b).*

**H5.**  *Having previously dissected pathological specimens, we hypothesize that the declawed felids will exhibit anomalous myological variation, i.e., qualitative differences in myological organization (e.g., combined or curtailed individual muscles) and/or greater intramuscular FL variation.*

## 3. Materials and Methods

### 3.1. Sample

We dissected the forearm muscles (Figure 1) of 18 felid specimens representing ten species that spanned nearly the entire body size range of the family (Table 1). Five of our specimens across almost all of this entire taxonomic and body size range were declawed early in the animals’ lives and many years prior to their deaths (Table 1). All of the specimens were given to us by zoos or a rescue facility, except for the wild bobcat (*Lynx rufus*; AHR 202146) specimen, which was obtained as a byproduct of the taxidermy industry. All captive animals were euthanized for reasons that did not pertain to their limbs or locomotor abilities, and no specimen died for the purpose of our study. All captive animals were kept in spaces that provided ample room for movement throughout the final years of their lives and were in generally good condition (neither emaciated nor obese) at the time of their euthanasia. The specimens were not declawed for this study; all declawed specimens were declawed prior to arriving at the rescue facility, where they all lived for many years subsequent to their onychectomies. While four of the five declawed specimens maintained relatively intact digital anatomy (e.g., onychectomies that follow Figure 2c,d), one specimen (*Panthera tigris*; 202152) had digital abnormalities that required medical intervention during the final years of its life, which was apparently a result of the declaw surgery; this case was likely an example of ungual process onychectomy (Figure 2b) and subsequent regrowth of remnant claw tissue (Figure 2e). Although this issue led to digital complications, the specimen did not have apparent forearm myological or osteological anomalies that made it noticeably different to other declawed specimens, and the unaffected forelimb was used for the study.

In addition to taking the mass and linear measurements of each muscle following previously described procedures [55], we directly measured muscle density (to the nearest 0.001 g/cm^3^) of all of the muscles small enough (e.g., each muscle from the smaller felids) to be accommodated using our Mettler Toledo density scale MS105 XPR-S XSR-S 0.1 mg, 1 mg density kit. For all muscle specimens for which density data were too large to enable direct measurement (i.e., from the larger specimens in the sample), we used muscle-specific density proxies measured using sections of each forearm muscle taken from one clawed *Panthera tigris* specimen (AHR 202157). The densities of the muscles of this specimen were measured by cutting sections out of the muscle to fit into the density scale; thus, this specimen was not used in the fascicle measurement procedure (since that process requires uncut fascicles). In addition to these myological measurements, species, sex, clawed status, and perimortem body mass were recorded. Where perimortem body mass was unavailable, species average body masses sourced from the literature [46] were used.

### 3.2. Architectural Variables Studied

Muscle fascicle architecture for all forearm muscles were included in this study. There is a great deal of individual variation in this anatomy, especially in, for instance, accessory digital extensor muscles that require a more detailed exploration of intra- and inter-specific variation in two other lineages [26]. Thus, while muscles that were conservative across the sample were analyzed individually, following our previous approaches [26,53,56], we also analyzed seven muscle combinations, despite individual muscle variation, to more broadly analyze muscle functional groupings: total forearm, total flexors, total extensors, wrist flexors, digital flexors, wrist extensors, and digital extensors.

All of the specimens were frozen once while fresh and thawed immediately prior to dissection, and no specimens showed signs of freezer damage. Little fiber shortening is expected, as specimens were frozen whole with skin on and the muscles intact and attached to the skeleton [57,58,59]. Only one forearm was studied for each specimen, and no specimen had excessive antemortem limb pathology (i.e., we did not dissect the forearm of the tiger limb with toe amputations).

Each specimen was skinned, and each forearm muscle was then excised using sharp dissection. Each muscle and its associated tendon was removed from its bony origin and insertion, except for the muscles that crossed the radiocarpal joint, in which case the tendons were severed at the distal radius following our previously described approach [26,53,55,56,60]. Muscle mass (MM) was recorded to the nearest 0.01 g for each muscle prior to chemical dissection. As noted above, density was also directly recorded for each muscle of eight of the smaller specimens (and recorded for the smaller muscles of a ninth specimen), and an additional larger tenth specimen was dissected and its muscles sectioned to calculate muscle-specific density proxies in combination with data from the six smaller clawed felids for which density data were measured.

Chemical dissection was performed following the approaches of Herrel et al. [61] and Boettcher et al. [56] to measure muscle architecture. In short, muscles were placed in 35% nitric acid at room temperature until the muscle fascicles were easily separated, ranging from twelve to fourteen hours, and they were then transferred to a 50% glycerin solution to prevent further breakdown [56]. Fascicles were separated from one another and photographed. A sample of approximately 40 representative fascicles per muscle was measured in ImageJ to establish the average fascicle length (FL) of each muscle. The physiological cross-sectional area (PCSA) of each individual muscle was calculated using MM and FL following Schumacher [62]:

*q* = *m*/*lp*

where *q* is PCSA (cm^2^), *m* is muscle mass, *l* is mean fiber length (cm), and *p* is muscle density.

Total MM, total PCSA, and weighted average FL were calculated for each of the seven functional groups described above. MM and PCSA were calculated by summing the MM or PCSA of all of the muscles in each functional group. A weighted average FL was calculated using the formula devised by Leischner [53]:

$$\frac{\sum_{n=1}^{i}{FL}_{n}\times {MM}_{n}}{\sum_{n=1}^{i}{MM}_{n}}$$

where *FL_n_* and *MM_n_* are the MM and FL of each muscle *n* in that group.

All analyses were performed in JMP Pro 17 (SAS) using a significance criterion of α = 0.05. The cube root of volume-related variables (body mass and MM) and the square root of area variables (PCSA) were taken, and all variables were logged to linearize and normalize the data. All myological variables for individual muscles and functional groups were separately RMA regressed against body mass for clawed and declawed specimens to assess and compare scaling. Residuals from the RMA line of the entire sample were saved for all individuals to allow us to adjust for body size. Analyses of variance (ANOVAs) were performed on these residuals to compare clawed and declawed myological variables.

To better represent the differences between clawed and declawed individuals, we compared theoretical clawed and declawed felids of the same body mass. Predicted myological variables were separately calculated using Ordinary Least Square (OLS) regression equations for clawed and declawed specimens for three different body masses: 10, 40, and 140 kg. Predicted values were reported as variables, where r^2^ ≥ 0.88 for regressions and *p* < 0.05 for ANOVAs. To compare theoretical clawed and declawed individuals at each body mass, proportions were calculated by dividing the declawed value by the clawed value for each body mass and multiplying the result by 100.

### 3.3. Qualitative Data

In addition to these quantitative measures and analyses, qualitative notes were taken, particularly to deduce observable trends related to differences in myological configuration between clawed and declawed specimens.

## 4. Results

### 4.1. Statistical Exclusion

As a consistent outlier (having consistently smaller myological variables than would be predicted based on its BM), the black-footed cat (*Felis nigripes*) specimen was excluded from statistical analysis. There are several possible explanations for the fact that this specimen may appear anomalous; for instance, it is possible that the specimen was in poorer condition (either emaciated antemortem or potentially slightly decomposed postmortem, though neither issue was evident on dissection). Also, as this was one of the specimens for which a species-specific BM was used instead of a specimen specific BM (which was not available at its donating facility), it is possible that this individual was much smaller than the average animal of its species and its myology was, therefore, not anomalously small, with the BM to which we ascribed it being incorrectly large. Regardless, as we did not want this single specimen—the smallest taxon in our sample and, therefore, a specimen that would have had an outsized effect on the regression lines—to skew what appeared to be the overarching biological pattern, it was excluded from the analyses. However, to ensure transparency, it is included in the figures. (That is, in JMP, the specimen was listed as “exclude”, but not as “hide”).

### 4.2. Allometry across the Sample

Overall, with one exception, forearm muscle mass (MM), physiological cross-sectional area (PCSA), and fascicle length (FL) scale with isometry or slight positive allometry against body mass for clawed felids were determined (Table 2).

For MM, most muscles scale isometrically or with positive allometry and correlate tightly (r^2^ = 0.91–0.99 for most muscles) with body mass (Table 2). Total flexors and digital flexors, which are mostly driven by FCU and FDP, scale with positive allometry. ECU and ECRL also scale with positive allometry. However, ECRB scales with slight negative allometry.

For PCSA, the distribution of isometric and positively allometric muscles is more evenly split and correlates less noticeably (r^2^ = 0.79–0.99 for most muscles) with body mass (Table 2). PCSA scales with positive allometry for total flexors and digital flexors, which are mostly driven by FDP. Wrist extensors, which are mostly driven by ECU, also scale with positive isometry, as do total forearm muscles, Sup., PQ, and FCU. For FL, all muscles scale isometrically, though, as has been shown for different lineages and muscle systems, the correlation between FL and BM is low (r^2^ = 0.26–0.79).

### 4.3. Differences between Clawed and Declawed Felid Forearms as a Whole

In many cases, declawed specimens scaled similarly to clawed felids. This observation is largely true across MM variables, with declawed MM largely effectively correlating with body mass (r^2^ = 0.88 to >0.99, other than FDS at 0.70; Table 2) and scaling in a similar manner to clawed felids. There is only one notable difference: digital flexors MM scale with positive allometry in clawed felids, while they scale with isometry in declawed felids. Declawed felid PCSA variables effectively correlate with body mass (r^2^ = 0.71 to >0.99; see Table 2) and largely scale in a similar manner to those of clawed felids; however, there are four instances in which PCSA shows positive allometry in clawed felids and isometry in declawed felids: total extensors, digital flexors, wrist extensors, and digital extensors. FL generally correlates better with body mass in declawed felids (r^2^ = 0.72–0.99, other than for digital flexors [0.18] and total flexors [0.24]). Total extensors and digital extensors scale with isometry in clawed specimens and slight positive allometry in declawed felids.

There were several instances of digital flexor muscles having significantly lower MM and PCSA in declawed felids (Table 3): MM was significantly lower in digital flexors (*p* < 0.001), and PCSA was significantly lower in total flexors (*p* < 0.0001) and digital flexors (*p* = 0.001).

**Figure 3:**
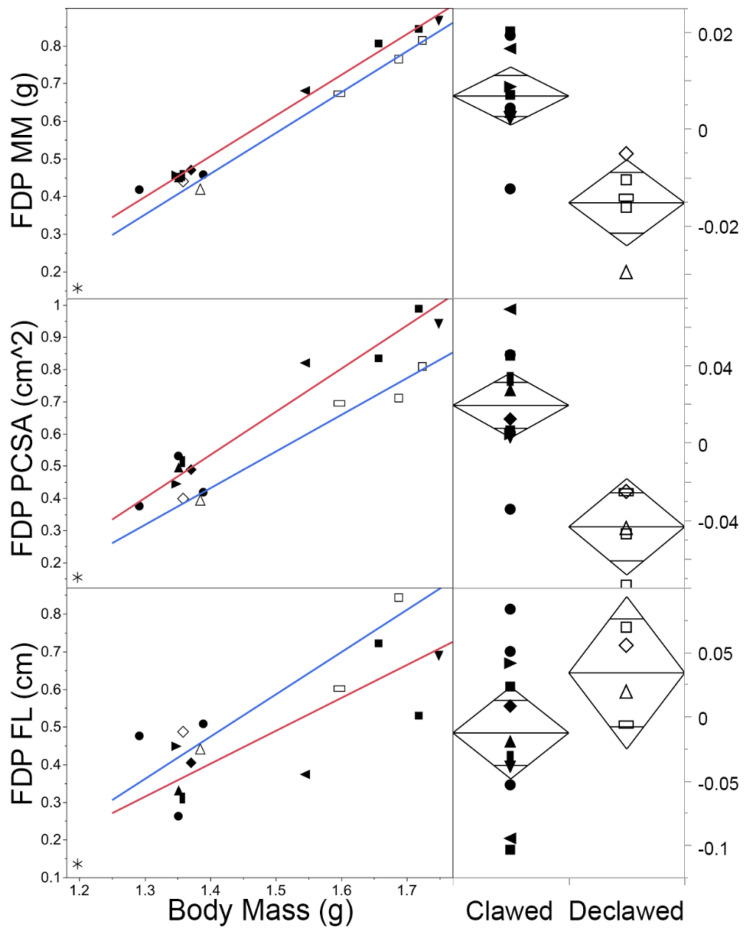
Reduced major axis regression of FDP MM, PCSA, and FL on BM and ANOVAs of residuals. All variables were linearized (i.e., volumetric and area variables taken to the cubic and square root) and logged prior to analysis. The red line represents the clawed (closed symbols) felid RMA regression, and the blue line represents the declawed (open symbols) felid RMA regression. Asterisk = *Felis nigripes* (excluded from analysis; see text); circle = *Leopardus pardalis*; upwards pointing triangle = *Lynx rufus*; right triangle = *Prionailurus viverrinus*; vertical rectangle = *Caracal caracal*; diamond = *Leptailurus serval*; horizontal rectangle = *Puma concolor*; left triangle = *Panthera pardus*; square = *P. tigris*; downwards triangle = *P. leo*. Declawed felids have significantly lower FDP MM and PCSA than clawed felids; results for FDP FL are not significantly different. Results are similar for FDS.

Using the OLS regression equations to compare theoretical clawed and declawed felids of the same sizes, we noted proportional differences in architectural variables with regression r^2^ > 0.88 and ANOVA *p* < 0.05 (Table 4): the MM for the reconstructed clawed specimen was larger than that of the reconstructed declawed specimen in several cases. The total MM of the flexors in theoretical declawed specimens was less than that of clawed specimens to the extent that the resulting total mass of the theoretical declawed specimens was 68.62–84.57% of clawed specimen, while for digital flexor MM, it was 52.54–65.07%. For both of these variables, the proportional differences were less extreme the larger the felid. Similar to MM, PCSA was often greater in clawed specimens than declawed specimens. For total forearm PCSA and digital extensors, the differences between clawed and declawed specimens were larger for larger hypothetical felids. For digital flexors, the differences were less extreme the larger the felid. For total flexors, there was little variation in proportion as body mass increased. The total forearm PCSA of declawed specimens is 71.57–79.32% that of the total forearm of the clawed specimen’s PCSA. For digital extensors, it was 66.13–87.11%; for total flexors, it was ~68.4% for all three body sizes; and for digital flexors specifically, declawed felids had merely 38.25–43.85% of the PCSA of clawed felids, which was substantially less than half of this force production proxy. These differences are evident in analyses of FDP across the sample more broadly (Figure 3).

### 4.4. Differences between Clawed and Declawed Felid Digital Muscles Specifically

For MM and PCSA, digital muscles scale similarly for clawed and declawed felids, except that MM and PCSA for FDP scale with positive allometry in clawed felids and isometry in declawed felids (Figure 3 top and middle). For FL, total extensor and digital extensors scale with positive allometry in declawed specimens and isometry in clawed specimens; however, this difference is not reflected in individual muscles (e.g., Figure 3 bottom). The average FL was higher in declawed specimens than clawed specimens for all analyzed digital muscles, though not to a statistically significantly extent.

**Figure 4:**
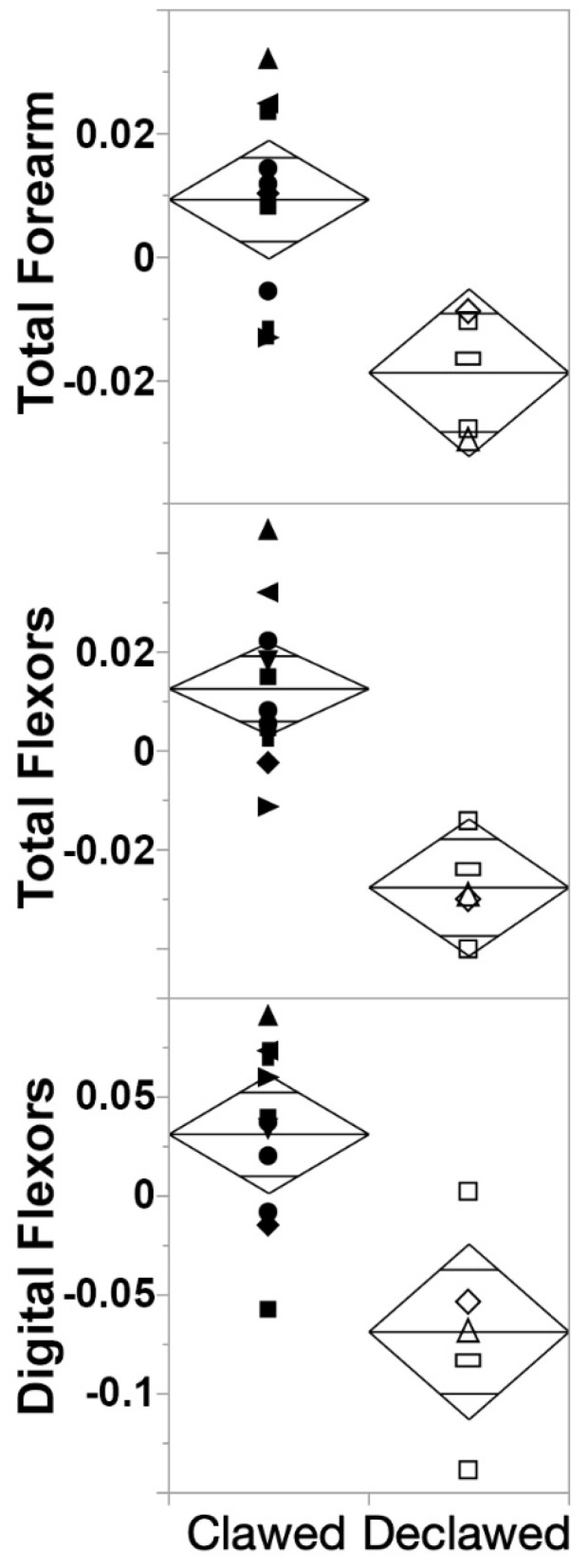
Residuals of flexor (total and digital) and total forearm PCSA. All variables were linearized and logged prior to analysis. Declawed felids have significantly lower digital and total flexors PCSA than clawed felids, and unlike H4b, the atrophy of these flexor muscles is not compensated for by other muscles, resulting in lower average (though statistically not significantly lower) forearm muscle PCSA (top). Results are similar for MM, though the reduction in the overall forearm MM of declawed felids is statistically significant. Asterisk = *Felis nigripes* (excluded from analysis; see text); circle = *Leopardus pardalis*; upwards pointing triangle = *Lynx rufus*; right triangle = *Prionailurus viverrinus*; vertical rectangle = *Caracal caracal*; diamond = *Leptailurus serval*; horizontal rectangle = *Puma concolor*; left triangle = *Panthera pardus*; square = *P. tigris*; downwards triangle = *P. leo*.

ANOVAs reveal that there were several instances of digital flexor muscles having significantly lower MM and PCSA in declawed felids (Table 3). MM was significantly lower in digital flexors (*p* < 0.01), being mostly driven by FDS (*p* = 0.02) and FDP (*p* < 0.01; Figure 3 top). PCSA (Figure 4 middle) was also significantly lower in digital flexors (*p* = 0.0013), being driven by FDS (*p* = 0.01) and FDP (*p* < 0.01; Figure 3 middle).

### 4.5. Differences between Clawed and Declawed Felid Wrist and other Non-Digital Forearm Muscles

For wrist muscles and other non-digital forearm muscles, there are some differences in scaling between clawed and declawed samples (Table 2). MM scales with positive allometry in clawed felids, whereas isometry scales in declawed felids in FCU, ECRL, and ECU. MM is isometric for clawed felids and positively allometric for declawed felids in BR, Sup., and PL, and it scales with negative allometry in clawed felids and isometry in declawed felids for ECRB.

For PCSA, there are also some differences in scaling between clawed and declawed samples (Table 2). PCSA scales with positive allometry in clawed felids and isometry in declawed felids in PQ and ECU, as well as with isometry in clawed felids and positive allometry in declawed felids in PT and ECR.

For FL, there is some positive allometry that is not found in clawed specimens, though only based on the functional group (Table 2). However, the average FL was higher in all declawed wrist muscles, other than ECRL, though not to a statistically significantly extent.

The MM and PCSA of some non-digital muscles were significantly lower in declawed specimens in total flexors, Sup., and ECRL MM and PCSA.

Differences between MM and PCSA of theoretical clawed and declawed felids of the same body mass can also be observed in the proportional values (Table 4). MM of the declawed specimens is 51.81–78.01% and 57.88–76.36% of that of clawed specimens for Sup. and ECRL, respectively. The PCSA of declawed specimens is 65.05–85.63% and 57.88–76.36% of that of clawed for Sup. and ECRL, respectively. For Sup., MM, and PCSA, the differences between clawed and declawed felids were less extreme the larger the felid, while the reverse was true for ECRL MM.

### 4.6. Anomalous Myological Qualitative Variation

In all of the complete amputation declawed specimens (i.e., Figure 2d), the deep digital flexor tendon was observed to be attached (presumably by scar tissue) to the next more proximal phalanx of each ray (i.e., the intermediate phalanges in rays II-V and the proximal phalanx in ray I). One specimen (declawed *P. tigris* 202152), which visually appeared to lack muscle mass on the anterior side of the forearm, had numerous fatty deposits and fluid-filled masses near joints throughout the forearm, wrist, and hand; accumulations of dense and hardened tissue in several tendons of the dissected limb; and in life-suffered ulcers, which led to further amputation of phalanges in the limb not used for the current study. It is possible that these major pathologies were the direct result of either its incomplete onychectomy (i.e., Figure 2b) and the resultant recurring regrowth (i.e., Figure 2e) or the fact that some were sequelae of general arthritis, which itself could have been exacerbated by the incomplete onychectomy.

## 5. Discussion

Onychectomy is common in domestic cats, being mostly performed to eliminate scratching of property and people [7,14]. While people are generally aware of this practice among domestic cats, fewer people may be aware that it has also been performed with some regularity on non-domestic species of felids [3,8,9,19]. Though the practice has been outlawed in many jurisdictions for domestic cats on ethical grounds [4,5], because of fundamentals of allometric scaling in anatomy (larger animals have a relatively greater body mass-to-foot surface area), onychectomy may have larger anatomical impacts on larger species. However, until now, no study had explored these effects. In the current study, we found clear trends in the scaling of the forearm muscles of both clawed and declawed felids, as well as significant anatomical differences between them.

### 5.1. Allometry across the Sample

Overall, for clawed felids, total forearm muscle mass (MM) scales had near-positive allometry (β = 1.12, confidence interval: 0.99–1.26). This result was driven by clear positive allometry of the flexors (in sum and digital, especially FDP; Table 2). This allometric trend was even clearer in the felid forearm physiological cross-sectional area (PCSA; Table 3). That is, the PCSA for the total forearm, total flexors, total extensors, digital flexors, and wrist extensors were all significantly positively allometric. This finding supports H1. While we predicted this outcome based on the allometric problem, it may also be driven by the fact that larger cats hunt large prey relative to their body size (e.g., servals predominantly hunt rodents, while some lions regularly hunt small elephants; [12]). Additionally, larger cats are relatively reliant on their forelimbs in terms of weight distribution and locomotor force [10,12]; thus, it is not surprising that the muscles in their forelimbs are relatively larger.

Overall, there is a consistent allometric trend in Sup., ECU, and FDP: in clawed animals, both the MM and PCSA related to these muscles scale with positive allometry relative to body mass (Table 2). The lack of a more universal trend may be due to the fact that not every muscle needs to be scaled up in relative size in order to overcome the allometric problem; as long as at least one muscle from a functional group is scaled up, it may be possible for the functional group as a whole to compensate for the scaling differences between large and small animals.

### 5.2. Differences between Clawed and Declawed Felid Forearms as a Whole

To assess if there is a difference between the impact of declawing on smaller and larger felids, we first needed to establish overall differences between clawed and declawed felids. Our first assessment of this type was based on RMA regressions of the myological variables and resulted in fewer of the lines for the declawed specimens scaling with positive allometry than those of the clawed specimens (Table 2 and Figure 3); however, this result is likely better explained by the smaller sample of declawed specimens and the greater myological variability within that sample than a functional difference. Perhaps because of the broader slope confidence intervals of the declawed specimens, there are no significant differences between the slopes of the declawed and clawed specimens. Thus, we cannot support our second hypothesis that onychectomy has a greater effect on relatively larger felids based on scaling. That myological variability might be explained by variations in the onychectomy method (Figure 2), developmental age at which the animals were declawed, length of time since the animals were declawed, or some other difference in their captive circumstances (e.g., animals kept in high quality sanctuaries or abusive situations for long periods prior to their arrival at a sanctuary).

There were substantial differences between clawed and declawed samples when comparing the residuals of several of the key variables via ANOVA (Table 3 and Figure 3 and Figure 4). Total flexor and digital flexor MM of clawed and declawed felids are significantly different, thus partially supporting our second hypothesis. The total forearm MM also approaches significant difference, which were, once again, driven by significant differences in the digital flexors. A similar trend occurs for PCSA, though total forearm PCSA differences are statistically significant. In all of these cases, the declawed specimens’ PCSAs are smaller than those of the clawed specimens (Figure 3). However, as the digital extensors were also inserted, in part, into the distal phalanx, we hypothesized that those muscles would also be significantly smaller in declawed felids, though this is not the case. Indeed, there was no significant effect of onychectomy on the myology of the digital extensors of felids, though the reduction in PCSA of declawed felids approaches statistical significance (Table 3).

As noted above, there is great variation in FL across our sample, including within the clawed and declawed felid subsamples. This kind of variation has been found in many studies of muscle fiber architecture, leading to a lack of resolution of non-isometric scaling trends, along with interesting variation related to functional adaptation [26,51,52,57,63]. In the current paper, although there are no statistically significant differences between the FL of the clawed and declawed subsamples, for most muscles and combinations thereof, the average FL of the declawed subsample is larger than that of the clawed subsample. As this trend is fairly consistent (87.5% of individual wrist and digital muscles), it seems likely that onychectomy leads to greater FL, though the variance seems to be statistically significant, while the effect size seems to be statistically insignificant. However, its functional effect may actually be large: this result could explain the “floppy foot” observed in declawed felids [3,45], as longer fascicles allow greater amounts of excursion, diminishing the rigidity required from the combined soft tissues to prevent postural collapse to the palmar surface [11,27,28]. While having longer fascicles is advantageous in, for instance, the masticatory muscles of taxa that consume large foods [57,63], this aspect could be the cause of the pathological laxity in the digits and wrists of declawed felids.

To further evaluate the differences between clawed and declawed felid myology across body size range (H2), we used OLS regression equations to compare the MM and PCSA of hypothetical clawed and declawed cats of 10, 40, and 140 kg in weight for which all variables were both highly correlated with body mass (r^2^ > 0.88) and the clawed and declawed subsamples differed significantly (Table 4). As noted above, declawed felids were smaller for all of these variables. However, contrary to H2, onychectomy did not consistently have a greater effect on relatively larger felids for all variables. For instance, the declawed MM for both digital flexors and total flexors was closer to that of clawed specimens the larger the animal (e.g., the MM for total flexors at 10 kg was 68.71% of that of clawed specimens, but at 140 kg wat 83.84%; Table 4). It is possible that this result occurred because larger animals cannot biomechanically afford to decrease their muscle mass too significantly, even when declawed; as they exert relatively greater weight through their forelimbs and are more dependent on them, they may not be able to experience as much atrophy in that region as small cats.

There were various trends in this analysis of PCSA: for total flexors, the proportion of PCSA of declawed hypothetical animals stayed consistent across body sizes; for digital flexors, declawed values more closely approached the clawed values the larger the animal; and for digital extensors and the total combined forearm, the PCSA of declawed animals differed more in larger felids than in small felids (Table 4). This result is not what we predicted, and it does not support our second hypothesis. While in the total forearm and digital extensors, the effects of onychectomy seem to be greater than in larger felids, partially supporting H2, this result did not hold true for digital flexors. This outcome is surprising as we inserted into the distal phalanges and predicted that they would, therefore, be the muscles most impacted by onychectomies. It is possible that a similar trend is happening in PCSA and MM: the larger animals simply cannot afford the reduction in force experienced by the smaller cats.

### 5.3. Differences between Clawed and Declawed Felid Digital Muscles

While the overarching allometric signals do not strongly differ between the clawed and declawed samples, the general trend in those lines may support our third hypothesis—the atrophy of the digital flexors—for one muscle: flexor digitorum profundus, which is the muscle most substantially related to the amputated distal phalanx. For that muscle, clawed felids trend more positively in PCSA slope than declawed felids (Figure 4), though (probably because of the small size of the declawed subsample) this difference is not statistically significant. As the slopes of their MM are nearly perfectly parallel (Figure 4; that said, declawed felids have significantly lower MM overall for this muscle and other muscles; see next section), the difference in PCSA slopes is driven entirely by differences in their fascicle lengths (Figure 4). That is, large and small felids have relatively similar FDP mass, while large declawed felids may have relatively lower FDP strength (the functional product of PCSA) because they seem to have relatively longer FDP fascicles. As is shown in Table 2 [51,52,57], there is so much variation in FL that it is difficult to deduce significant scaling for this or any muscle, which have no fewer statistical differences in FL scaling.

Although we have not statistically deduced whether larger declawed felids are myologically more pathological than smaller declawed felids, it is clear that onychectomy has a strongly significant effect on forearm myology across the sample as a whole: declawed felids have significantly smaller values for both mass and PCSA of their FDS and FDP than clawed felids (Table 3, Figure 3 and Figure 4). Although most of the other forearm muscles do not differ between clawed and declawed felids, as mentioned above, the differences in FDS and FDP MM and PCSA are so significant that they lead to significant differences between combined digital flexors and total flexors (Figure 4). This outcome supports our third hypothesis, and the trend is likely explained by the fact that these digital flexors are the muscles that have tendons most directly impacted by onychectomy.

For the single muscle most greatly affected by onychectomy, i.e., the FDP (the tendon of which normally inserts into the amputated distal phalanx), the regression lines are so parallel that our hypothetical declawed felids have ~73% MM of clawed felids across their entire body size range, while, contrary to H2, relative MM increases for hypothetical declawed felids, as they grow for their combined total flexors and digital flexors. This finding could relate to the increased need for postural support in larger felids. That is, because of the allometric problem, larger felids might need to maintain forearm MM to support their body weight and more front limb-reliant behaviors, while smaller declawed felids might be more atrophied [10,12]. However, the PCSA of FDP is reduced as body mass increases. This finding, once again, may relate to the statistically small change in FL that produces a functionally large impact. While the mass of FDP is not significantly relatively reduced in larger cats, the increased laxity of the muscles causes a decrease in PCSA and, thus, a less functionally powerful FDP.

### 5.4. Differences between Clawed and Declawed Felid Wrist and Other Non-Digital Forearm Muscles

The scaling of non-digital felid forearm muscles varies greatly between clawed and declawed specimens. However, once again, this outcome is likely caused by the relatively small sample size of declawed specimens, as well as variance in onychectomy approaches (Figure 2).

For most of the non-digital forearm muscles, there is not a significant difference in either MM- or PCSA-differentiated clawed and declawed felids. However, in partial support of H4a, the muscles of declawed felids were generally lower in MM and PCSA than those of the clawed felids, with this difference being significant for Sup. and ECRL (Table 3). It is unclear why these two muscles would be more substantially affected by onychectomy than other non-digital forearm muscles, though this result is potentially related to general variation in muscle architecture within our sample.

Contrary to H4b, none of the forearm muscles of declawed felids are relatively larger. Thus, although their digital flexors are significantly reduced, contrary to that sub-hypothesis, none of the other muscles apparently compensate for that reduction. This result also contributes to the reduction in overall forearm MM and PCSA discussed above.

The MM of ECRL is smaller in declawed cats than in clawed cats, and this difference is more extreme as the body mass increases. However, for Sup., the opposite trend is found for MM and PCSA. While they are smaller in declawed cats than in clawed cats, supporting the fourth hypothesis, these opposing trends are perplexing [12].

### 5.5. Anomalous Myological Variation

The lack of notable qualitative differences in the myology of declawed specimens is surprising. Although the sample size of declawed specimens is small, making it difficult to observe both quantitative and qualitative trends, given the radical osteological effect of this surgery—which partially or completely removes phalanges—and the fact that this surgery targets a distal limb element that is the sole attachment of one of the major locomotor muscles, we anticipated seeing dramatic visual differences, including major rearrangements of the tendons and other digital locomotor muscle bellies (e.g., to compensate for the FDP) in the muscles beyond the relatively simple atrophy that we observed and quantified (even the FDP tendon of the declawed specimens—the tendon of the muscle most directly affected by this surgery—simply seems to attach to the next most proximal structures). In fact, the qualitative variation that we observed was distributed across the whole sample—both clawed and declawed animals—and most evident in the digital extensors, as was observed in the forearms of other lineages [26]. The only relevant trend that was noted was that the deep digital flexor tendon of declawed animals was attached to the intermediate phalanx of each ray—probably through scarification of the tissue—as would be most expected for any functional remnant of this muscle.

## 6. Conclusions

There are alternatives to declawing domestic cats that are considered more humane and better for the welfare of animals. If onychectomy is elected, many vets suggest only declawing the front claws to reduce pain and preserve certain behaviors, such as scaling trees [42]. However, many of these alternatives are either entirely unavailable or not as successful in non-domestic cats. Regardless, this research shows that onychectomy has a substantial effect on the myology of both small and large cats. It dramatically affects the deep digital flexor—the muscle that attaches specifically to the phalanges that are amputated in the procedure—as well as other muscles. Furthermore, the other forearm muscles do not compensate for the losses in these most affected muscles; declawed felids have weaker forearms and are biomechanically compromised beyond merely removing the targeted claws. While this practice is questionable in for domestic cats (and is indeed, for this reason, illegal in many places), onychectomy has even more substantial biomechanical effects on larger felids and may, therefore, be considered as an even crueler practice in these non-domestic species.

In the future, this study could be expanded upon through a detailed analysis of the osteological effects of onychectomy (e.g., how does declawing change the cross-sections of the remaining weight-bearing elements?) and the use of three-dimensional myological visualization techniques, like Diffusible Iodine-Based Contrast-Enhanced Computed Tomography (diceCT), which would allow the visualization of muscles in three dimensions, potentially including their individual fascicular structures, as they attach to bones. Not only could diceCT confirm the results presented in this study, but it could also give more insight into variables that were difficult to examine through traditional dissection methods, such as muscle reorganization (combined or curtailed individual muscles) and differences in origins and/or insertions. Unfortunately, diceCT currently does a poor job of differentiating between collagenous structures; thus, it is not ideal for the visualization of tendons and ligaments, both of which would be vital to improve understanding relative to onychectomy. While Magnetic Resonance Imaging (MRI) does a better job regarding the visualization of these kinds of structures, the most accessible devices do not currently have sufficiently high resolution (i.e., small enough voxel sizes) to substantially add to our current understanding of this anatomy. However, as accessible MRI resolutions improve or new staining regimes that allow better visualization of collagenous soft-tissues emerge, these technologies would allow us to have an even more detailed understanding of the morphological effects of onychectomy in exotic felids.

## Figures and Tables

**Table 1:** Sample.

Species (Common Name)	Specimen ^a^	Sex	Claw Status	Body Mass (kg)
*Caracal caracal* (Caracal)	202148	Female	Clawed	11.79
*Felis nigripes* (Black-footed cat)	202143	Unknown	Clawed	3.90 ^b^
*Leopardus pardalis* (Ocelot)	202136	Female	Clawed	11.30
*Leopardus pardalis* (Ocelot)	202147	Male	Clawed	7.48
*Leopardus pardalis* (Ocelot)	202137	Male	Clawed	14.70
*Leptailurus serval* (Serval)	202140	Male	Clawed	12.93
*Lynx rufus* (Bobcat)	202146	Male	Clawed	11.34
*Panthera leo* (Lion)	202150	Male	Clawed	176.40
*Panthera pardus* (Leopard)	202141	Male	Clawed	43.09
*Panthera tigris* (Tiger)	202134	Female	Clawed	93.44
*Panthera tigris* (Tiger)	202144	Female	Clawed	142.88
*Panthera tigris* (Tiger) ^c^	202157	Female	Clawed	140.16
*Prionailurus viverrinus* (Fishing cat)	202149	Unknown	Clawed	11.05 ^b^
*Leptailurus serval* (serval)	202135	Male	Declawed	11.88
*Lynx rufus* (bobcat)	202105	Male	Declawed	14.24
*Puma concolor* (cougar)	202145	Male	Declawed	61.69
*Panthera tigris* (tiger)	202127	Female	Declawed	115.21
*Panthera tigris* (tiger)	202152	Male	Declawed	147.87

^a^ AHR catalog number of the collection at North Carolina State University. We contacted author AHR to gain access to (including via loan) osteological remains associated with these (and other) specimens. ^b^ Species average body mass—all other body masses are specific to the dissected individual; ^c^ specimen used for density measurement, rather than fiber architecture analysis.

**Table 2:** RMA scaling for MM, PCSA, and FL for functional groups, as well as individual muscles for clawed and declawed specimens.

		MM					PCSA					FL				
Muscle	Sample	Y-Intercept	Slope (β)	Lower β CL	Upper β CL	r^2^	Y-Intercept	Slope (β)	Lower β CL	Upper β CL	r^2^	Y-Intercept	Slope (β)	Lower β CL	Upper β CL	r^2^
Total forearm	Clawed	−0.86	1.12	0.99	1.26	0.98	−0.93	1.32 ^a^	1.17 ^a^	1.50	0.98	−0.90	0.93	0.53	1.60	0.73
	Declawed	−0.95	1.16	0.95	1.41	0.99	−0.89	1.25 ^a^	1.06 ^a^	1.49	0.99	−1.01	1.00	0.45	2.24	0.88
Total flexors	Clawed	−0.95	1.12 ^a^	1.02 ^a^	1.22	0.99	−1.10	1.36 ^a^	1.22 ^a^	1.51	0.98	−0.80	0.77	0.41	1.47	0.64
	Declawed	−1.10	1.19 ^a^	1.03 ^a^	1.38	0.99	−1.18	1.35 ^a^	1.16 ^a^	1.56	0.99	−0.96	0.88	-	-	0.24
Total extensors	Clawed	−1.03	1.12	0.96	1.31	0.97	−1.32	1.39 ^a^	1.05 ^a^	1.85	0.90	−0.74	0.84	0.44	1.60	0.67
	Declawed	−0.97	1.07	0.79	1.45	0.97	−0.89	1.08	0.73	1.61	0.96	−1.10	1.12 ^a^	1.09 ^a^	1.15	1.00
Wrist flexors	Clawed	−1.17	1.15	0.87	1.52	0.88	−1.33	1.38	0.96	1.99	0.82	−1.45	1.10	0.44	2.73	0.52
	Declawed	−1.12	1.13	0.70	1.81	0.95	−1.05	1.20	0.79	1.83	0.96	−1.71	1.30	0.69	2.42	0.92
Digital flexors	Clawed	−1.05	1.14 ^a^	1.02 ^a^	1.28	0.98	−1.45	1.49 ^a^	1.12 ^a^	1.99	0.88	−0.84	0.85	0.25	2.92	0.44
	Declawed	−1.27	1.23	0.84	1.80	0.96	−1.81	1.60	0.61	4.17	0.86	−1.05	1.03	-	-	0.18
Wrist extensors	Clawed	−1.11	1.13	0.93	1.38	0.95	−1.54	1.47 ^a^	1.02 ^a^	2.12	0.85	−0.68	0.83	0.36	1.93	0.59
	Declawed	−0.99	1.05	0.81	1.38	0.98	−1.00	1.11	0.80	1.54	0.97	−0.97	1.05	0.92	1.19	1.00
Digital extensors	Clawed	−1.11	1.06	0.96	1.17	0.98	−1.24	1.16	0.94	1.44	0.93	−1.12	1.05	0.70	1.57	0.79
	Declawed	−1.21	1.11	0.74	1.69	0.96	−1.07	1.01	0.53	1.90	0.91	−1.55	1.37 ^a^	1.03 ^a^	1.82	0.98
BR	Clawed	−1.34	1.14	0.89	1.46	0.91	−2.03	1.38	0.84	2.27	0.73	−0.91	1.31	0.46	3.71	0.48
	Declawed	−1.77	1.39 ^a^	1.08 ^a^	1.79	0.98	−1.80	1.17	0.78	1.75	0.96	−1.93	1.96	0.92	4.19	0.89
Sup.	Clawed	−1.53	1.21	0.99	1.48	0.94	−1.48	1.25 ^a^	1.13 ^a^	1.37	0.98	−1.82	1.25	0.72	2.16	0.70
	Declawed	−1.84	1.37 ^a^	1.10 ^a^	1.70	0.99	−2.10	1.57 ^a^	1.20 ^a^	2.07	0.98	−1.36	0.97	0.70	1.35	0.97
PT	Clawed	−1.02	0.96	0.75	1.22	0.91	−1.22	1.12	0.97	1.29	0.97	−0.96	0.84	0.16	4.27	0.40
	Declawed	−1.31	1.15	0.96	1.36	0.99	−1.49	1.27 ^a^	1.05 ^a^	1.54	0.99	−1.05	0.93	0.48	1.81	0.91
PQ	Clawed	−1.32	1.07	0.95	1.21	0.98	−1.46	1.26 ^a^	1.07 ^a^	1.49	0.95	−1.47	0.98	0.42	2.29	0.54
	Declawed	−1.60	1.25	0.88	1.78	0.97	−1.47	1.27	0.93	1.73	0.97	−1.88	1.23	0.72	2.09	0.93
FCR	Clawed	−1.16	1.01	0.74	1.39	0.86	−1.21	1.04	0.62	1.77	0.71	−1.79	1.42	0.63	3.21	0.56
	Declawed	−1.29	1.09	0.86	1.39	0.98	−1.27	1.07	0.79	1.43	0.98	−1.41	1.18	0.78	1.78	0.96
FCU	Clawed	−1.38	1.24 ^a^	1.06 ^a^	1.46	0.96	−1.64	1.52 ^a^	1.21 ^a^	1.91	0.93	−1.32	0.99	0.36	2.69	0.53
	Declawed	−1.36	1.22	0.82	1.81	0.96	−1.29	1.29 ^a^	1.01 ^a^	1.65	0.98	−1.99	1.41	-	-	0.77
PL	Clawed	−1.65	1.40	0.41	4.74	0.83	−2.05	1.77	-	-	0.67	−1.76	1.29	-	-	0.50
	Declawed	−1.56	1.32 ^a^	1.06 ^a^	1.63	1.00	−1.89	1.59	0.50	5.09	0.93	−1.34	1.05	-	-	0.72
FDS	Clawed	−2.13	1.66	0.69	3.95	0.57	−2.94	2.25	0.39	13.09	0.43	−1.19	0.92	0.59	1.43	0.79
	Declawed	−2.91	2.00	0.16	25.61	0.78	−4.12	2.73	-	-	0.71	−0.83	0.73	0.29	1.85	0.86
FDP	Clawed	−1.01	1.08 ^a^	1.00 ^a^	1.17	0.99	−1.34	1.34 ^a^	1.10 ^a^	1.62	0.94	−0.82	0.87	0.35	2.17	0.52
	Declawed	−1.06	1.08	0.90	1.30	0.99	−1.16	1.14	0.81	1.59	0.97	−1.10	1.12	-	-	0.86
ECU	Clawed	−1.29	1.14 ^a^	1.01 ^a^	1.28	0.98	−1.63	1.45 ^a^	1.16 ^a^	1.82	0.93	−1.40	1.03	-	-	0.32
	Declawed	−1.43	1.21	0.89	1.66	0.97	−1.62	1.41	0.84	2.35	0.94	−1.24	0.93	0.38	2.31	0.87
ECRL	Clawed	−1.45	1.27 ^a^	1.08 ^a^	1.48	0.98	−2.07	1.54	0.85	2.79	0.82	−0.70	1.03	-	-	0.35
	Declawed	−1.35	1.16	0.89	1.51	0.99	−1.85	1.31 ^a^	1.10 ^a^	1.56	1.00	−0.39	0.87	0.44	1.72	0.96
ECRB	Clawed	−0.75	0.75 ^b^	0.60	0.93 ^b^	0.97	−1.12	0.99	0.53	1.85	0.81	−0.83	0.87	-	-	0.26
	Declawed	−0.99	0.95	0.37	2.42	0.94	−1.04	0.90	-	-	0.79	−1.19	1.19	0.56	2.53	0.96

^a^ indicates slopes of muscles that scale with significant positive allometry. ^b^ indicates slopes of muscles that scale with significant negative allometry. Abbreviations: BR = m. brachioradialis; SUP = m. supinator; PT = m. pronator teres; PQ = m. pronator quadratus; FCR = m. flexor carpi radialis; FCU = m. flexor carpi ulnaris; PL = m. pollicis longus; FDS = m. flexor digitorum superficialis; FDP = m. flexor digitorum profundus; ECU = m. extensor carpi ulnaris; ECRL = m. extensor carpi radialis longus; ECRB = m. extensor carpi radialis brevis.

**Table 3:** ANOVA analyses that compare MM and PCSA between clawed and declawed felids. FL is not included as none indicated significant differences between clawed and declawed felids.

Muscle	*p*-Value (MM)	*p*-Value (PCSA)	F-Ratio (MM)	F-Ratio (PCSA)	DF
Total forearm	0.054	0.003 *	4.48	13.31	1, 13
Total flexors	0.005 *	<0.001 *	11.03	26.67	1, 14
Total extensors	0.585	0.372	0.31	0.85	1, 13
Wrist flexors	0.629	0.870	0.24	0.03	1, 14
Digital flexors	<0.001 *	0.001*	17.96	16.03	1, 14
Wrist extensors	0.890	0.744	0.02	0.11	1, 13
Digital Extensors	0.288	0.052	1.22	4.52	1, 14
BR	0.135	0.164	2.52	2.15	1, 14
Sup.	0.030 *	<0.001 *	5.84	31.17	1, 14
PT	0.934	0.148	0.01	2.35	1, 14
PQ	0.794	0.884	0.07	0.02	1, 14
FCR	0.884	0.537	0.02	0.40	1, 14
FCU	0.706	0.970	0.15	<0.01	1, 13
PL	0.564	0.290	0.37	1.31	1, 7
FDS	0.017 *	0.012 *	7.53	8.58	1, 13
FDP	<0.001 *	0.001 *	19.52	20.12	1, 14
ECU	0.235	0.182	1.55	1.99	1, 13
ECRL	0.012 *	0.011 *	9.33	10.09	1, 10
ECRB	0.320	0.510	1.11	0.47	1, 9

* indicates *p*-values of <0.05. Abbreviations: BR = m. brachioradialis; SUP = m. supinator; PT = m. pronator teres; PQ = m. pronator quadratus; FCR = m. flexor carpi radialis; FCU = m. flexor carpi ulnaris; PL = m. pollicis longus; FDS = m. flexor digitorum superficialis; FDP = m. flexor digitorum profundus; ECU = m. extensor carpi ulnaris; ECRL = m. extensor carpi radialis longus; ECRB = m. extensor carpi radialis brevis.

**Table 4:** Proportional Differences (100 × declawed/clawed) between MM and PCSA. Empty cells and all FL proportions are not shown because of the lack of significant scaling in these variables.

Category	Body Mass (kg)	Proportion MM	Proportion PCSA
Total forearm	10		79.32
	40		75.15
	140		71.57
Total flexors	10	68.62	68.35
	40	76.58	68.38
	140	84.57	68.41
Digital extensors	10		87.11
	40		75.37
	140		66.13
Digital flexors	10	52.54	38.25
	40	58.79	41.10
	140	65.07	43.85
SUP	10	50.65	43.37
	40	65.51	58.33
	140	82.65	76.23
ECRL	10	76.04	
	40	66.74	
	140	59.32	
FDP	10	72.59	65.53
	40	72.91	55.69
	140	73.21	48.08

Abbreviations: SUP; m. supinator, ECRL; m. extensor carpi radialis longus, FDP; m. flexor digitorum profundus.

## Data Availability

Raw data and osteological remains of all specimens used in this study are available upon request from the corresponding author.
